# Three New Compounds from *Aspergillus terreus* PT06-2 Grown in a High Salt Medium

**DOI:** 10.3390/md9081368

**Published:** 2011-08-12

**Authors:** Yi Wang, Jinkai Zheng, Peipei Liu, Wei Wang, Weiming Zhu

**Affiliations:** Key Laboratory of Marine Drugs, Chinese Ministry of Education, School of Medicine and Pharmacy, Ocean University of China, Qingdao 266003, China; E-Mails: wangyi@hotmail.com (Y.W.); zhjk8212@yahoo.cn (J.Z.); liupeipei@ouc.edu.cn (P.L.); wwwakin@ouc.edu.cn (W.W.)

**Keywords:** *Aspergillus terreus*, high salinity metabolites, terremides A and B, terrelactone A

## Abstract

To investigate the structurally novel and bioactive natural compounds from marine-derived microorganisms under high salinity, the fungus *Aspergillus terreus* PT06-2 was isolated from the sediment of the Putian Sea Saltern, Fujian, China. Three new compounds, terremides A (**1**) and B (**2**) and terrelactone A (**3**), along with twelve known compounds (**4**–**15**) were isolated and identified from the fermentation broth of *A. terreus* PT06-2 at 10% salinity. Among these metabolites, compounds **4** and **15** only produced in the 10% salinity culture, were identified as methyl 3,4,5-trimethoxy-2-(2-(nicotinamido) benzamido) benzoate, and (+)-terrein, respectively. The new compounds **1** and **2** exhibited antibacterial activity against *Pseudomonas aeruginosa* and *Enterobacter aerogenes* with MIC values of 63.9 and 33.5 μM, respectively. Compounds **5** showed moderate anti-H1N1 activity and lower cytotoxicity with IC_50_ and CC_50_ values of and 143.1 and 976.4 μM, respectively.

## 1. Introduction

Solar salterns matured a surprisingly rich diversity and abundance of halophilic and halotolerant fungi [1]. Furthermore, some genes could be activated at high salt concentrations; some new secondary metabolites were probably produced by halotolerant fungi. During pursing the halotolerant microbes from hypersaline ecological niches [2–6], *Aspergillus terreus* PT06-2 was isolated and identified from the sediment collected in the Putian Salterns, Fujian, China. *A. terreus* is commonly isolated from cultivated or non-cultivated soils with worldwide distribution and was first published in 1918 [7]. The famous statins drug lovastatin, an inhibitor of 3-hydroxy-3-methylglutaryl-coenzyme A reductase (HMG-CoA reductase), for lowering cholesterol to prevent cardiovascular disease, is mainly produced by *A. terreus* [8,9]. A series of compounds such as terreineol [10], terreulactone A [11], terrain [12], terreic acid [13] and aspulvinones [14] were also isolated from this fungus. Chemical screening for *A. terreus* PT06-2 showed that the chemical diversity of the secondary metabolites is the richest at 10% salinity compared with those at 0% and 3% salt. Chemical investigation in 10% salinity resulted in isolation and identification of three new compounds, terremides A and B (**1**, **2**), terrelactone (**3**), along with twelve known compounds (**4**–**15**) [15–26]. Antibacterial activity of the new compound **2** against *Enterobacter aerogenes*, and the new compound **1** and **4** against *Pseudomonas aeruginosa* were observed. Compound **5** showed moderate anti-H1N1 activity and lower cytotoxicity.

## 2. Results and Discussion

### 2.1. The Identification of New Metabolites from *Aspergillus terreus* PT06-2 at 10% Salinity

**Figure 1.**
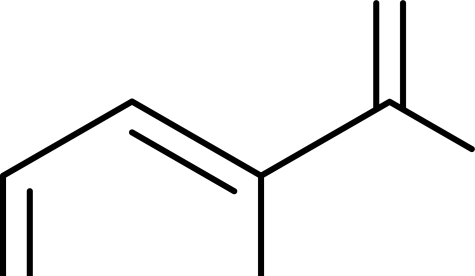
Chemical structures of the metabolites (**1**–**15**) from *A. terreus* PT06-2.

Fungus *A. terreus* PT06-2 was incubated in a high-salt medium containing 10% salt and extracted with EtOAc to afford a crude extract. The crude extract (31.2 g) was separated by extensive chromatography using silica gel, Sephadex LH-20 and HPLC to give compounds **1**–**15** (Figure 1), including three new compounds: terremides A and B (**1**, **2**), terrelactone (**3**), and twelve known ones: methyl 3,4,5-trimethoxy-2-(2-(nicotinamido)benzamido)benzoate (**4**) [15], (+)-butyrolactones I–III (**5**–**7**) [16–19], 3-hydroxy-5-[[4-hydroxy-3-(3-methyl-2-buten-1-yl)phenyl]methyl]-4-(4-hydroxyphenyl)-2(5*H*)-furanone (**8**) [20], aspernolide A (**9**) [16], 5-[(3,4-dihydro-2,2-dimethyl-2*H*-1-benzopyran-6-yl)-methyl]-3-hydroxy-4-(4-hydroxyphenyl)-2(5*H*)-furanone (**10**) [18], territrem B (**11**) [22], (−)-(1*R*,4*R*)-1,4-(2,3)-indomethane-1-methyl-2,4-dihydro-1*H*-pyrazino[2,1-*b*]quinazoline-3,6-dione (**12**) [23], *R*(−)-6-hydroxymellein (**13**) [24], *trans*-4,6-dihydroxymellein (**14**) [25], and (+)-terrein (**15**) [26].

**Figure 2.**
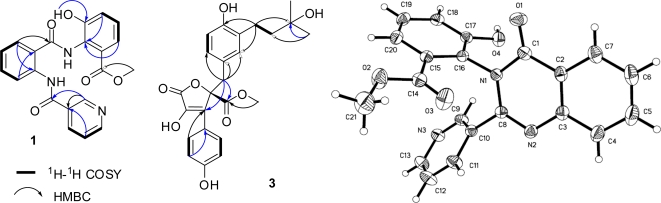
The key COSY and HMBC correlations for compounds **1** and **3**, and the final X-ray drawing of compound **2**.

**Terremide A** (**1**) gave an HRESIMS peak at *m/z* 392.1257 [M + H]^+^ (calcd for C_21_H_18_N_3_O_5_, 392.1246), corresponding to the molecular formula C_21_H_17_N_3_O_5_. The UV spectrum displayed absorption at λ_max_ 205 and 262 nm, similar to that of compound **4** [15]. The 1D NMR spectra were also similar to those of **4**, indicating the same molecular skeleton [15]. The differences were a 1,2,3-trisubstituted benzene nucleus instead of the 1,2,3,4,5-pentasubstituted one, and the corresponding signals of three methoxy groups did not display in the 1D NMR spectra of **1**. Besides, the downfield shifts for C-1″–C-3″ were observed, suggesting **1** as the derivative of **4** by demethoxylation at C-4″ and C-5″, and demethylation at 3″-MeO. This deduction was further supported by the key ^1^H-^1^H COSY correlations of H-4″/H-5″/H-6″, and the key HMBC correlations (Figure 2) form HO-3″ (δ_H_ 12.22) to C-3″ (δ_C_ 149.8), from H-4″ (δ_H_ 7.70) and H-6″ (δ_H_ 7.36) to C-2″ (δ_C_ 128.2), and from CH_3_O- (δ_H_ 3.95) to carbonyl carbon (δ_C_ 169.2). Thus, structure of **1** was determined to be methyl 3-hydroxy-2-(2-(nicotinamido)benzamido)benzoate.

**Terremide B** (**2**) gave an HRESIMS peak at *m/z* 374.1160 [M + H]^+^ (calcd for C_21_H_16_N_3_O_4_, 374.1141), corresponding to the molecular formula C_21_H_15_N_3_O_4_ that required 16 degrees of unsaturation. Compared with compound **1**, the molecular formula of **2** is one H_2_O less and one more degree of unsaturation than that of **1**. Both **1** and **2** showed similar ^1^H and ^13^C NMR spectra, except for the disappearance of two exchangeable proton signals at δ_H_ 11.96/9.08, and the replacement of the amido carbon signal at δ_C_ 163.7 by a quaternary carbon signal at δ_C_ 147.2. Furthermore, another amido carbon signal shifted upfield from 169.1 ppm to 160.7 ppm, revealing existence of a quinazolinone nucleus [27]. Thus, compound **2** was deduced as the dehydrated and cyclized product of **1**. When reacted in MeOH, compound **1** formed **2** in 96% yields. Therefore, the structure of terremide B (**2**) was assigned as methyl 3-hydroxy-2-(4-oxo-2-(pyridin-3-yl)quinazolin-3(4*H*)-yl)benzoate that was further determined by X-ray single-crystal diffraction analysis (Figure 2).

**Terrelactone** (**3**) gave an HRESI-MS peak at *m/z* 465.1529 [M + Na]^+^ (calcd for C_24_H_26_O_8_Na, 465.1525), corresponding to the molecular formula C_24_H_26_O_8_. Its UV spectrum showed characteristic absorption of butyrolactones at 231 and 310 nm [21]. 1D NMR showed signals of a 1,2-disubstituted benzene nucleus at δ_H_ 7.62 (2H, d, 8.2)/6.97 (2H, d, 8.2), a 1,2,4-trisubstituted benzene nucleus at δ_H_ 6.55 (d, 8.2)/6.51 (brs)/6.52 (d, 8.2), a carbomethoxy at δ_H/C_ 3.78/53.7/170.9, and an unsaturated butyrolactone at δ_C_ 86.0/132.6/139.0/168.7, suggesting the same skeleton as butyrolactone I (**5**) [18,19]. The NMR differences between **3** and **5** were that the signals belonging to the CH═C double band at δ_H/C_ 5.06/122.4/131.4 in **5** were replaced by a methylene signal at δ_H/C_ 1.55/44.4 and a quaternary carbon signal at δ_C_ 70.2 (Table 1). These data suggested that compound **3** was the hydrated derivative of **5** at CH═C double band. This assignment was further supported by ^1^H-^1^H COSY correlation between H-1‴ and H-2‴, and HMBC correlations from H-1‴ to C-2′, C-4′ and C-3‴, from H-4‴/5‴ to C-2‴ and C-3‴ (Figure 2). The dextral specific rotation ([α]^25^_D_ +71) indicated *R*-configuration at C-4 [17]. Thus, the structure of terrelactone (**3**) was determined as (*R*)-methyl 4-hydroxy-2-(4-hydroxy-3-(3-hydroxy-3-methylbutyl)benzyl)-3-(4-hydroxyphenyl)-5-oxo-2,5-dihydrofuran-2-carboxylate.

### 2.2. The Effects of Salt Stress on Production of Secondary Metabolites from *Aspergillus terreus* PT06-2

**Figure 3.**
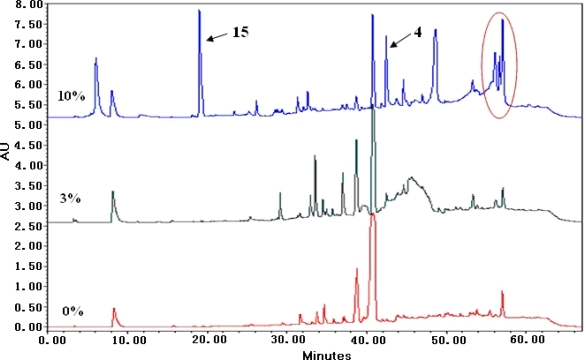
HPLC profiles of secondary metabolites from *A. terreus* PT06-2 cultured in different salt conditions (0%, 3% and 10%, respectively). Peaks circled were red pigments (HPLC eluent: 0–60 min, 5–100% CH_3_OH; flow rate: 1 mL/min).

Salt-tolerant fungi belong to extremophiles which can survive under the conditions of zero to high salinity. However, using salt-tolerant fungi to produce new secondary metabolites at high salinity was rarely reported [2–6]. In order to investigate the effect of high salt stress on fungal secondary metabolites, *A. terreus* PT06-2 was cultured at 0%, 3% and 10% salt. The amount of EtOAc extracts of metabolites in 10% salinity was the largest (222 mg *vs.* 165 and 200 mg of 0% and 3% salinity). The chemical diversities of the metabolites in 10% salinity were increased (Figure 3). Compounds **4** and **15** were not produced by *A. terreus* PT06-2 when cultivated in 0% and 3% salinity media. The other sole secondary metabolites were a kind of red pigments (Figure 3) whose structures were not identified.

### 2.3. The Bioactivities of Metabolites from *Aspergillus terreus* PT06-2 in 10% Salinity

The new compounds (**1**–**3**), sole metabolites (**4**, **15**) produced at 10% salinity, and typical butyrolactones (**5**–**10**) of *A. terreus* were evaluated for their cytotoxicity against HL-60 and BEL-7402 cell lines, antimicrobial activity against *Enterobacter aerogenes*, *Pseudomonas aeruginosa*, *Staphylococcus aureus* and *Candida albicans* (Table 2), and antiviral activity against influenza virus (H1N1) by MTT [28], agar dilution method [29], and CPE inhibition assay [30,31], respectively. Compound **5** showed weak cytotoxicity against HL-60 with an IC_50_ (half maximal inhibitory concentration) value of 57.5 μM. Compounds **1**, **4** and **2** showed weak antibacterial activity against *S. aureus* and *E. aerogenes* with MIC (minimum inhibitory concentration) of 63.9, 52.4 and 33.5 μM, respectively (Table 2). Other compounds did not show cytotoxicity and antibacterial activity (IC_50_ or MIC > 100 μM). Butyrolactone I (**5**) showed anti-H1N1 activity with IC_50_ and CC_50_ (50% cytotoxicity concentration) values of 143.1 and 976.4 μM, respectively (positive control: ribavirin, IC_50_ 100.8 μM). In addition, it was reported that butyrolactone I (**5**) showed kinase inhibition with high selectivity towards CDK1 and CDK2 [32].

## 3. Experimental Section

### 3.1. General Experimental Procedures

Optical rotations were obtained on a JASCO P-1020 digital polarimeter. UV spectra were recorded on a Beckman DU 640 spectrophotometer. IR spectra were obtained on a Nicolet NEXUS 470 spectrophotometer as KBr disks. ^1^H NMR, ^13^C NMR, and DEPT spectra and 2D-NMR were recorded on a JEOL JNMECP 600 spectrometer using TMS as internal standard, and chemical shifts were recorded as δ values. ESIMS was measured on a Q-TOF Ultima Global GAA076 LC mass spectrometer. Semipreparative HPLC was performed using an ODS column (YMC-pack ODS-A, 10 × 250 mm, 5 μm, 4 mL/min). HPLC was performed using an ODS column (YMC-pack C18, 4.6 × 250 mm, 5 μm, 2 mL/min). TLC and column chromatography (CC) were performed on plates precoated with silica gel GF254 (10–40 μm) and over silica gel (200–300 mesh, Qingdao Marine Chemical Factory, Qingdao, China) and Sephadex LH-20 (Amersham Biosciences, Sweden), respectively. All the materials for the culture medium of *A. terreus* PT06-2 was purchased from Qingdao Marine Chemical Factory, Qingdao, China.

### 3.2. Fungal Material

The fungus *A. terreus* PT06-2 was isolated from sediment (saline 20%), Putian Saltern of Fujian Province of China. It was identified according to its morphological characteristics and analyses of its 18S rRNA sequence (Genbank JN006059) by Prof. C. X. Fang, China Center for Type Culture Collection. A voucher specimen is deposited in our laboratory at −80 °C. The working strain was prepared on potato dextrose agar slants and stored at 4 °C.

### 3.3. Fermentation and Extraction

The fungus *A. terreus* PT06-2 was grown under static conditions at 28 °C for 35 days in 100 1000-mL conical flasks containing liquid medium (300 mL/flask, pH 7.0) composed of glucose (10 g/L), maltose (20 g/L), mannitol (20 g/L), monosodium glutamate (10 g/L), yeast extract (3 g/L), corn steep liquor (1 g/L), and 10% salt (NaCl 8%, MgSO_4_ 0.5%, KH_2_PO_4_ 0.5%, NH_4_Cl 0.5% and KCl 0.5%). The fermented whole broth (30 L) was filtered through cheesecloth to separate the supernatant from the mycelia. The supernatant was concentrated under reduced pressure to about 5 L and then extracted three times with EtOAc to give an EtOAc solution, while the mycelia were extracted three times with acetone. The acetone was removed under reduced pressure to afford a residual aqueous solution. This aqueous solution was extracted three times with EtOAc to give a further EtOAc crude extract. Both EtOAc solutions were combined and concentrated under reduced pressure to give an extract (31.2 g).

The fungus *A. terreus* PT06-2 was incubated under the same conditions in 0%, 3% and 10% salt. The chemical diversities of the secondary metabolites of EtOAc extract were investigated with HPLC.

### 3.4. Purification

The extract (31.2 g) from *A. terreus* PT06-2 was separated into ten fractions (Fraction 1 to 10) on a silica gel column using a step gradient elution with CHCl_3_–petroleum ether (0–100%) and then with MeOH–CHCl_3_ (0–100%). Fraction 4 was separated on Sephadex LH-20 with MeOH–CHCl_3_ (1:1) and 100% MeOH to obtain Fraction 4-2 and 4-3. Fraction 4-2 was further purified on semipreparative HPLC (60% MeOH) to give compound **15** (106 mg, *t*_R_ 12 min); Fraction 4-3 was separated on a silica gel column using a step gradient elution with Me_2_CO–petroleum (0–100%) to obtain Fraction 4-3-1 and 4-3-2. Fraction 4-3-1 was purified on semipreparative HPLC (60% MeOH) to give compound **13** (7 mg, *t*_R_ 8 min). Fraction 5 was separated on silica gel column using a step gradient elution with Me_2_CO–petroleum (0–100%) to obtain fraction 5-1 and fraction 5-5. Fraction 5-1 was rechromatographed on silica gel column (MeOH–CHCl_3_, 1:50), Sephadex LH-20 (MeOH–CHCl_3_, 1:1), and then semipreparative HPLC (70% MeOH) to give compound **4** (87 mg, *t*_R_ 8 min), **11** (21 mg, *t*_R_ 12 min) and **12** (7 mg, *t*_R_ 14 min). Fraction 5-5 was chromatographed on Sephadex LH-20 (MeOH-CHCl_3_, 1:1), and then semipreparative HPLC (40% MeOH) to give compound **14** (2 mg, *t*_R_ 9 min). Fraction 8 was separated on Sephadex LH-20 (MeOH-CHCl_3_, 1:1) twice to obtain Fraction 8-2 and 8-4. Fraction 8-2 was chromatographed on Sephadex LH-20 (100% MeOH), and then semipreparative HPLC (60% MeOH) to give compound **5** (200 mg, *t*_R_ 23 min), **7** (10 mg, *t*_R_ 12 min), and **8** (5 mg, *t*_R_ 18 min). Fraction 9 was separated on silica gel column (MeOH–CHCl_3_, 1:10) to obtain Fraction 9-1–9-4. Fraction 9-1 was further purified on semipreparative HPLC (70% MeOH) to give compound **1** (6 mg, *t*_R_ 10 min), **9** (70 mg, *t*_R_ 12 min), and **10** (4 mg, *t*_R_ 10 min). Fraction 9-3 was chromatographed on ODS column (50% MeOH), and then semipreparative HPLC (60% MeOH) to give compound **2** (3 mg, *t*_R_ 6 min), **6** (3 mg, *t*_R_ 15 min). Fraction 9-5 was chromatographed on ODS column (40% MeOH), and then semipreparative HPLC (60% MeOH) to give compound **3** (45 mg, *t*_R_ 10 min).

**Terremide A** (**1**): white amorphous powder; UV (MeOH) λ_max_ (log €) 205 (4.4), 262 (3.2) nm; ^1^H and ^13^C NMR data, see Table 1; HRESIMS *m/z* 392.1257 [M + H]^+^ (calcd for C_21_H_18_N_3_O_5_, 392.1246).

**Terremide B** (**2**): colorless crystal; UV (MeOH) λ_max_ (log €) 212 (4.4), 283 (3.3) nm; ^1^H and ^13^C NMR data, see Table 1; HRESIMS *m/z* 374.1160 [M + H]^+^ (calcd for C_21_H_16_N_3_O_4_, 374.1174).

**Terrelactone** (**3**): pale yellow oil; [α]^25^_D_ +71 (*c* 0.5, CHCl_3_); UV (MeOH) λ_max_ (log €) 231 (4.0), 310 (4.4) nm; IR (KBr) ν_max_ 3275, 2932, 1744, 1703, 1604, 1527, 1433, 1380, 1225, 1146, 839 cm^−1^; ^1^H and ^13^C NMR data, see Table 1; HRESIMS *m/z* 465.1529 [M + Na]^+^ (calcd for C_24_H_26_O_8_Na, 465.1525).

### 3.5. Chemical Transformation of **1** into **2**

Compound **1** (2 mg, 5 μmol) was dissolved in MeOH (0.5 mL) and stirred for 24 h at room temperature. The reaction mixture was purified by semi-preparative HPLC eluting with 70% CH_3_OH to give compound **2** (1.8 mg, 96% yield) as colorless crystals.

### 3.6. X-ray Crystal Structure of **2**

Compound **2** was obtained as colorless monoclinic crystals with molecular formula C_21_H_15_N_3_O_4_. Space group C2/c, *a* = 27.130 (3) Å, *b* = 8.9484 (13) Å, *c* = 14.6147 (17) Å, α = 90.00°, β = 91.248 (2)°, γ = 90.00°, *V* = 3547.2 (8) Å^3^, *Z* = 8, crystal size 0.45 × 0.40 × 0.29 mm^3^. A total of 3131 unique reflections (2θ < 50°) were collected on a CCD area detector diffractometer with graphite monochromated MoKa radiation (λ = 0.71073 Å). The structure was solved by direct methods (SHELXS-97) and expanded using Fourier techniques (SHELXL-97). The final cycle of full-matrix least squares refinement was based on 3131 unique reflections (2θ < 50°) and 254 variable parameters and converged with unweighted and weighted agreement factors of *R*_1_ = 0.0679, *R*_w_ = 0.1074 and *R* = 0.0366 for *I* > 2sigma(*I*) data. Crystallographic data (excluding structure factors) for structure **2** in this paper have been deposited with the Cambridge Crystallographic Data Centre as supplementary publication number CCDC 819714. Copies of the data can be obtained, free of charge, on application to CCDC, 12 Union Road, Cambridge CB2 1EZ, UK (Fax: +44-(0)-1223-336033 or E-Mail: deposit@ccdc.cam.ac.uk).

### 3.7. Bioassay

Antimicrobial activity against *E. aerogenes*, *P. aeruginosa*, *S. aureus* and *C. albicans* was evaluated using an agar dilution method [29]. The tested strains were cultivated in LB agar plates for bacteria and in YPD agar plates for *C. albicans*, at 37 °C. Compounds and positive controls were dissolved in 5% DMSO-H_2_O at different concentrations from 1000 to 62.5 μg/mL and then from 50 to 0.78 μg/mL, using continuous 2-fold dilution. The test solutions (5 μL) were absorbed onto paper disks (5 mm diameter) and placed on the assay plates. After 24 h incubation, zones of inhibition (mm in diameter) were recorded. The minimum inhibitory concentrations were defined as the lowest concentration at which an inhibition zone could be observed.

Cytotoxicity against HL-60 and BEL-7402 cancer cell lines and confluent MDCK cells was assayed by the MTT method [28]. Cell lines were grown in RPMI-1640 supplemented with 10% FBS under a humidified atmosphere of 5% CO_2_ and 95% air at 37 °C. Cell suspensions, 200 μL, at a density of 5 × 10^4^ cell mL^−1^ were plated in 96-well microtiter plates and incubated for 24 h. Then, 2 μL of the test solutions (in MeOH) were added to each well and further incubated for 72 h. The MTT solution (20 μL, 5 mg/mL in IPMI-1640 medium) was then added to each well and incubated for 4 h. Old medium containing MTT (150 μL) was then gently replaced by DMSO and pipetted to dissolve any formazan crystals formed. Absorbance was then determined on a SPECTRA MAX PLUS plate reader at 540 nm. The CC_50_ was calculated as the compound concentration necessary to reduce cell viability by 50%. Vp-16 (Etoposide) was used as the positive control with IC_50_ values of 0.042 and 0.83 μM, respectively.

The antiviral activity against H1N1 was evaluated by the CPE inhibition assay [30,31]. Confluent MDCK cell monolayers were firstly incubated with influenza virus (A/Puerto Rico/8/34 (H1N1), PR/8) at 37 °C for 1 h. After removing the virus dilution, cells were maintained in infecting media (RPMI 1640, 4 μg/mL of trypsin) containing different concentrations of test compounds at 37 °C. After 48 h incubation at 37 °C, cells were fixed with 100 μL of 4% formaldehyde for 20 min at room temperature. After removal of the formaldehyde, the cells were stained with 0.1% crystal violet for 30 min. The plates were washed and dried, and the intensity of crystal violet staining for each well was measured in a microplate reader (Bio-Rad, USA) at 570 nm. The IC_50_ was calculated as the compound concentration required inhibiting influenza virus yield at 48 h post-infection by 50%. Ribavirin was used as the positive control with the IC_50_ values of 100.8 μM.

## 4. Conclusions

In summary, fifteen metabolites including three new compounds were isolated and identified from the fermentation broth of *A. terreus* PT06-2 under 10% salinity conditions. High salt stress affected the quantity and profile of secondary metabolites. The new compounds **1** and **2**, and the sole metabolite **4** produced under high salt stress conditions showed antibacterial activity. The typical metabolite of *A. terreus*, butyrolactone I (**5**), showed anti-H1N1 activity with low cytotoxicity, indicating that **5** might be a promising drug candidate for anti-influenza virus.

## Supporting Information



## Figures and Tables

**Table 1.** ^1^H- and ^13^C-NMR (600 and 150 MHz) data for compounds **1**–**3** in CDCl_3_.

**Position**	**1^a^**		**2**		**3^b^**	

	**δ_C_**	**δ_H_(*J*in Hz)**	**δ_C_**	**δ_H_(*J*in Hz)**	**δ_C_**	**δ_H_(*J*in Hz)**
1	119.1, qC		160.7, qC		168.7, qC	
2	140.3, qC		153.4, qC		139.0, qC	
3	122.0, CH	8.87, d (8.3)	147.2, qC		132.6, qC	
4	134.2, CH	7.67, t (7.8)	127.4, CH		86.0, qC	
5	124.0, CH	7.32, t (7.8)	134.8, CH		170.9, qC	
6	128.1, CH	7.99, d (7.8)	127.3, CH	7.79, d (7.9)	39.1, CH_2_	3.44, s
7	169.1, qC		126.4, CH	7.92, t (7.9)		
8			120.6, qC	7.61, t (7.9)		
9			131.3, qC	8.16, d (7.9)		
10			147.9, CH			
1′	130.3, qC		150.1, CH		124.8, qC	
2′	148.8, CH	9.26, s	122.5, CH	8.57, s	132.6, CH	6.51, br s
3′					128.2, qC	
4′	152.6, CH	8.78, d (4.2)	135.0, CH	8.48, d (4.5)	154.8, qC	
5′	123.6, CH	7.45, t “like” (6.0)	160.7, qC	7.29, dd (4.5, 7.9)	115.1, CH	6.52, br d (8.2)
6′	134.9, CH	8.27, d (6.0)	153.4, qC	7.75, d (7.9)	129.4, CH	6.55, d (8.2)
7′	163.7, qC					
1″	120.1, qC		124.4, qC		122.8, qC	
2″	128.2, qC		128.7, qC		130.1, CH	7.62, br d (8.2)
3″	149.8, qC		153.5, qC		116.5, CH	6.97, br d (8.2)
4″	123.5, CH	7.70, d (7.8)	121.2, CH	7.03, d (7.8)	158.7, qC	
5″	126.7, CH	7.26, t (7.7)	130.0, CH	7.28, t (7.8)	116.5, CH	6.97, br d (8.2)
6″	126.3, CH	7.36, d (8.2)	120.0, CH	7.33, d (7.8)	130.1, CH	7.62, br d (8.2)
7″	169.2, qC		165.2, qC			
7″-OMe	53.0, CH_3_	3.95, s	52.2, CH_3_	3.65, s		
2-NH		11.96, s				
2″-NH		9.08, s				
3″-OH		12.22, s				

a The NMR data for 2-NH, 2″-NH and 3″-OH are δ_H_ 11.96 (s), 9.08 (s) and 12.22 (s), respectively;

b Recorded in acetone-*d*_6_. The NMR data for isoprenyl moiety and 5-OCH_3_ are δ_H_ 2.55 (m)/2.45 (m), 1.55 (2H, m), 1.18 (6H, s), and 3.78 (3H, s), and δ_C_ 25.3 (CH_2_), 44.4 (CH_2_), 70.2 (CH), 29.8 × 2 (CH_3_), and 53.7 (CH_3_), respectively.

**Table 2.** Antimicrobial activities of compounds **1**–**4** (“–” indicates not measured).

**Compound**	**MIC (μM)**			
	***Enterobacter aerogenes***	***Pseudomonas aeruginosa***	***Staphylococcus aureus***	***Candida albicans***
**1**	>100	>100	63.9	>100
**2**	33.5	>100	>100	>100
**3**	>100	>100	>100	>100
**4**	>100	>100	52.4	>100
**15**	>100	>100	>100	>100
Ciprofloxacinlactate	1	30	1	−
Ketoconazole	−	−	−	5
