# Supplementary material for: Three New Compounds from Aspergillus terreus PT06-2 Grown in a High Salt Medium
Source: Mar Drugs. 2011 Aug 12;9(8):1368–78. doi: 10.3390/md9081368 (PMC3164379; doi:10.3390/md9081368)

Supporting Information

Three New Compounds from *Aspergillus terreus* PT06-2 Grown in a High Salt Medium

Yi Wang, Jinkai Zheng, Peipei Liu, Wei Wang and Weiming Zhu *

Key Laboratory of Marine Drugs, Chinese Ministry of Education, School of Medicine and Pharmacy, Ocean University of China, Qingdao 266003, China

Figure S1. The ^{1}H-NMR spectrum of compound **1** in $CDCl_3$ S2
Figure S2. The ^{13}C-NMR spectrum of compound **1** in $CDCl_3$ S3
Figure S3. The ^{1}H-NMR spectrum of compound **2** in $CDCl_3$ S4
Figure S4. The ^{13}C-NMR spectrum of compound **2** in $CDCl_3$ S5
Figure S5. The ^{1}H-NMR spectrum of compound **3** in acetone-d_6 S6
Figure S6. The ^{13}C-NMR spectrum of compound **3** in acetone-d_6 S7

Figure S1. The ^{1}H-NMR spectrum of compound **1** in $CDCl_3$.

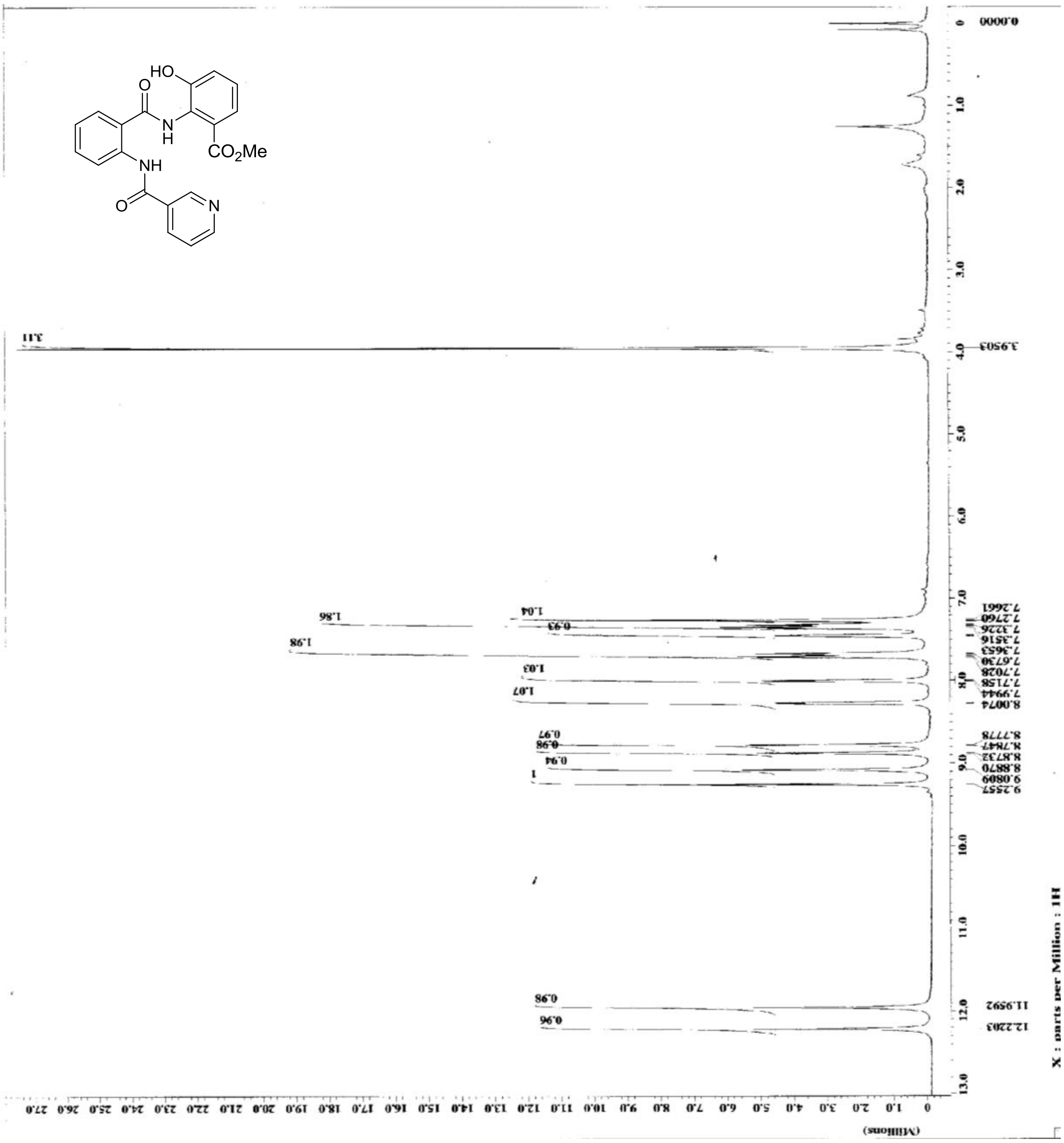

Figure S2. The ^{13}C-NMR spectrum of compound **1** in $CDCl_3$.

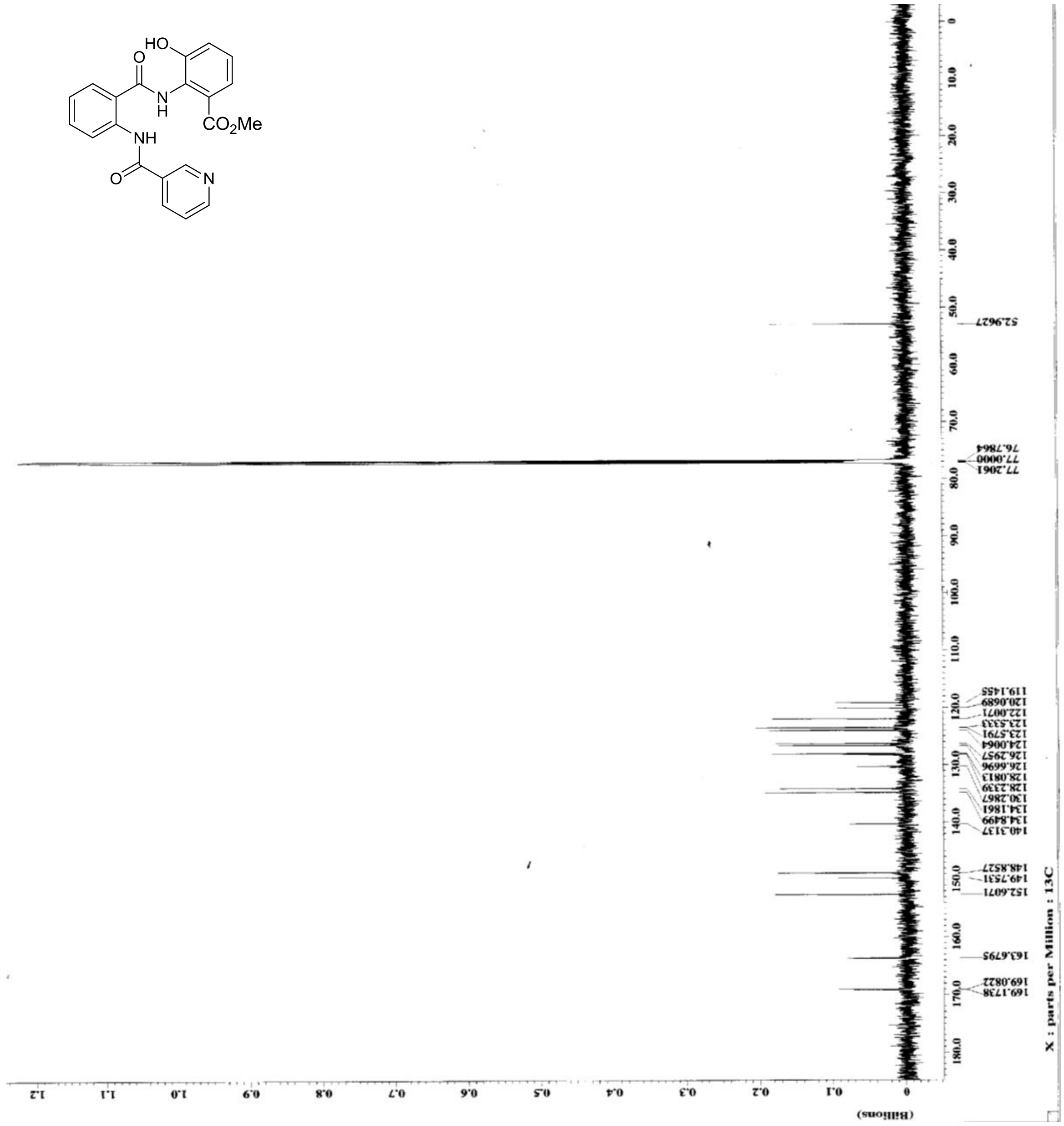

Figure S3. The ^{1}H-NMR spectrum of compound **2** in $CDCl_3$.

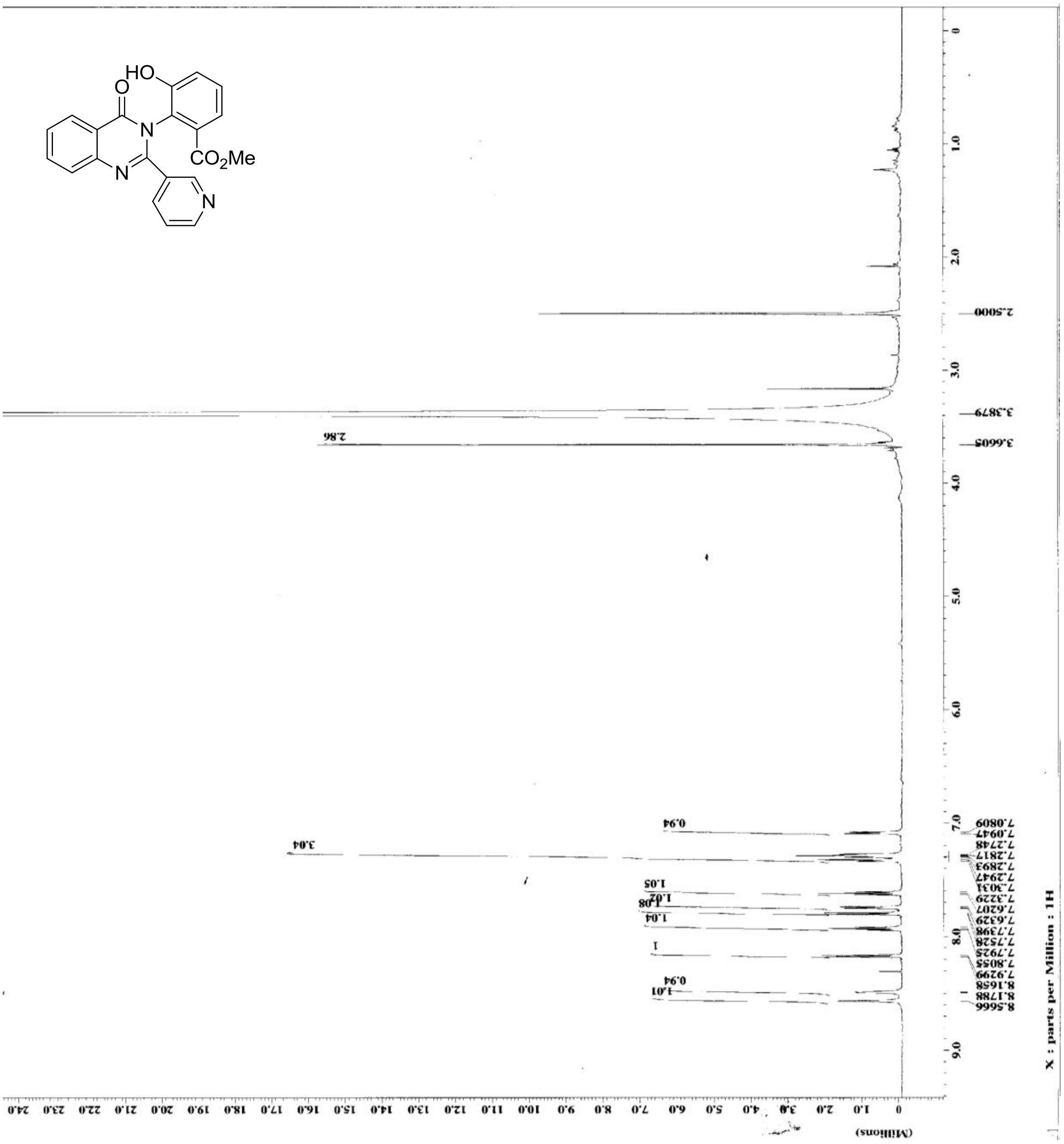

Figure S4. The ^{13}C-NMR spectrum of compound **2** in $CDCl_3$.

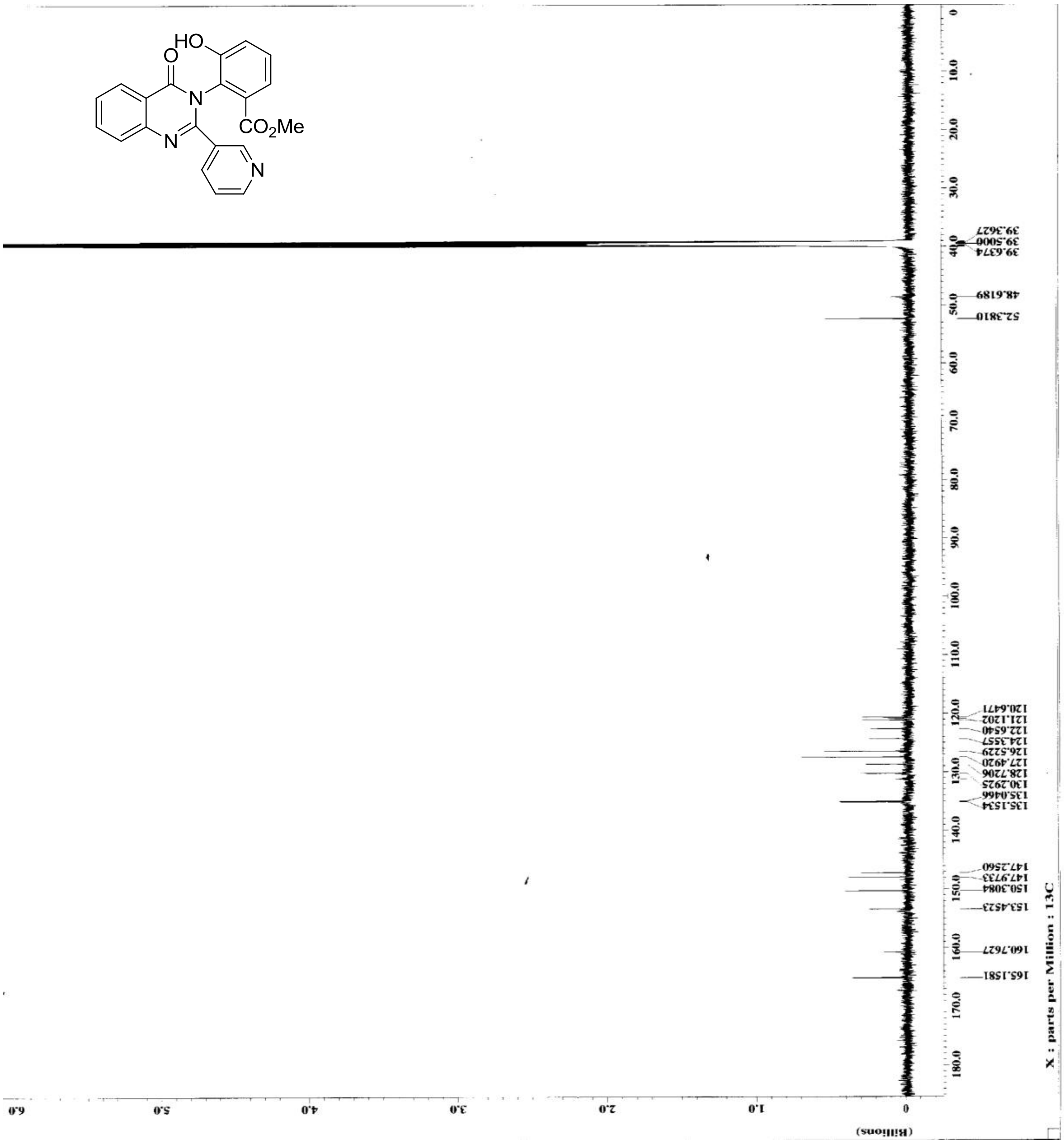

Figure S5. The ^{1}H-NMR spectrum of compound **3** in acetone-d_6.

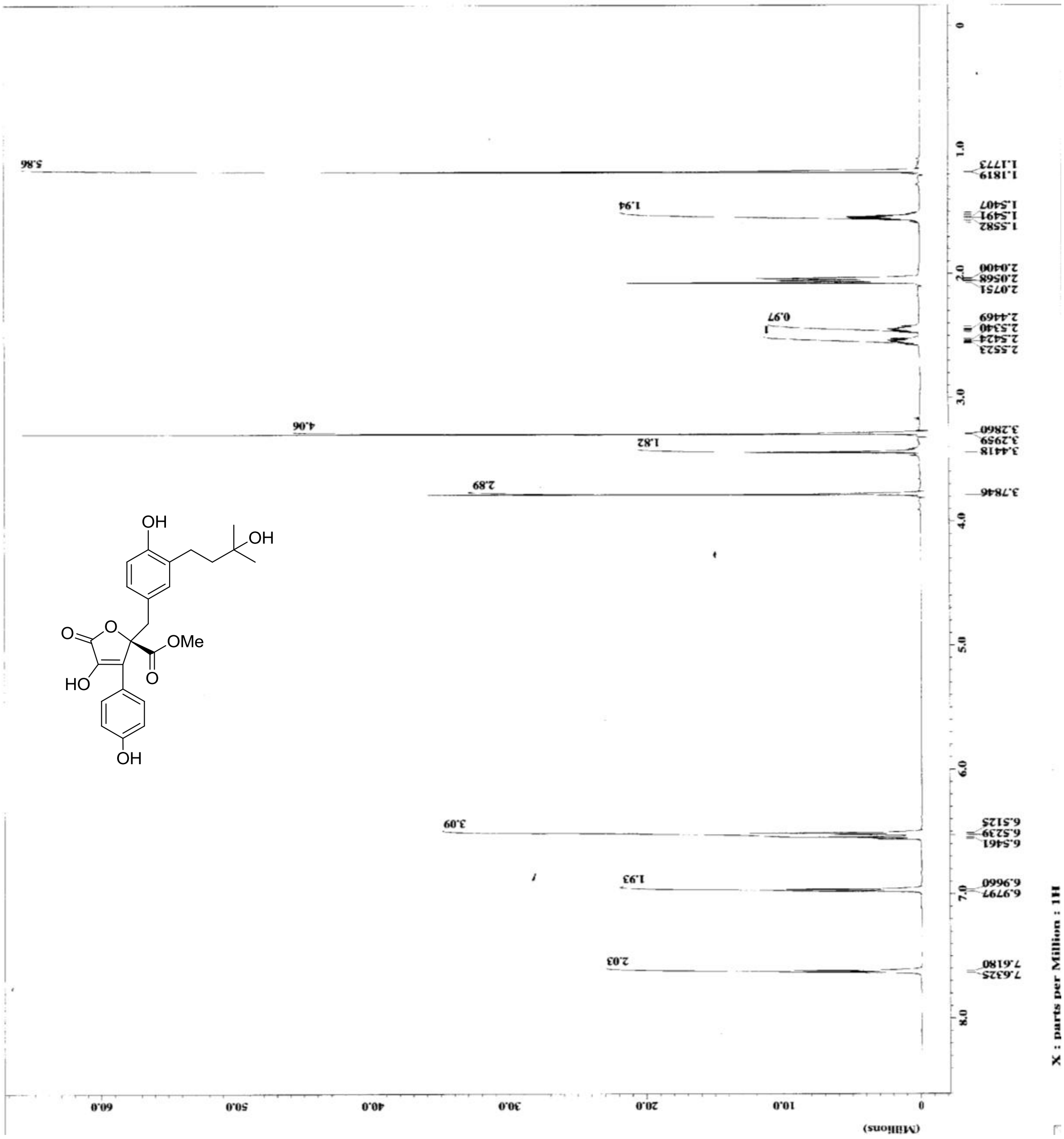

Figure S6. The ^{13}C-NMR spectrum of compound **3** in acetone-d_6.

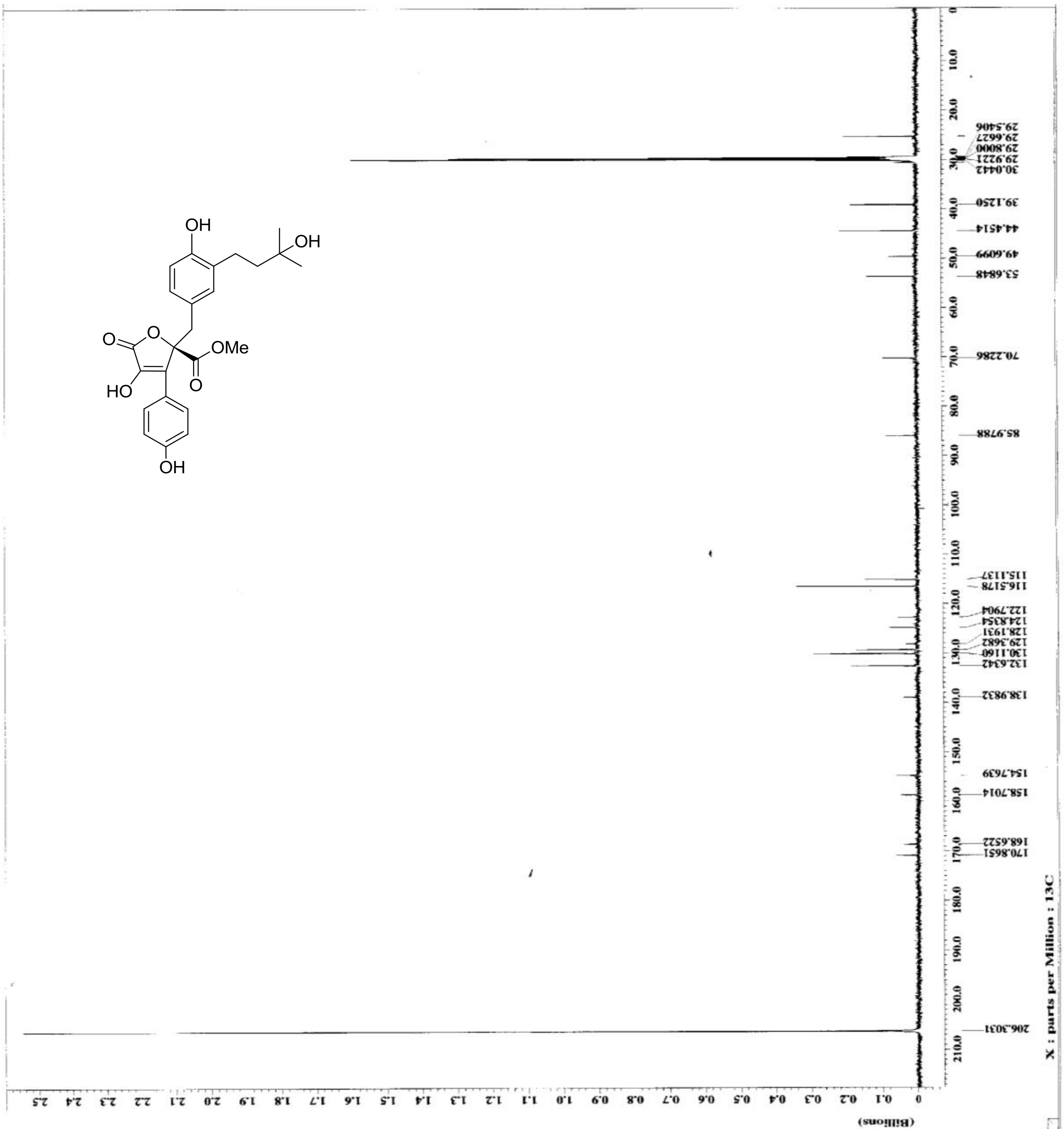

Supplement: Supplementary file 1 [file marinedrugs-09-01368-s001.pdf]
